# Supplementary material for: Overview of current state of research on the application of artificial intelligence techniques for COVID-19
Source: PeerJ Comput Sci. 2021 May 26;7:e564. doi: 10.7717/peerj-cs.564 (PMC8176528; doi:10.7717/peerj-cs.564)
Supplement: Supplemental Information 1 [file peerj-cs-07-564-s001.docx]

**Table 1.** Symptoms associated with COVID-19 patients

| **Symptom** | **Symptoms in COVID-19 patients (in %)** |
| --- | --- |
| Fever | 98 |
| Cough | 76 |
| Fatigue | 44 |
| Sputum Production | 28 |
| Headache | 8 |
| Haemoptysis | 5 |
| Diarrhoea | 3 |
