# Supplementary material for: Overview of current state of research on the application of artificial intelligence techniques for COVID-19
Source: PeerJ Comput Sci. 2021 May 26;7:e564. doi: 10.7717/peerj-cs.564 (PMC8176528; doi:10.7717/peerj-cs.564)
Supplement: Supplemental Information 2 [file peerj-cs-07-564-s002.docx]

**Table 2**. Estimation of severity of COVID-19 patients.

| **Age**  **(in years)** | **Symptom cases that requires**  **hospitalization (in %)** | **Hospitalization cases that requires critical care (in %)** | **Infection Fatality Ratio** |
| --- | --- | --- | --- |
| 0-9 | 0.1 | 5.0 | 0.002 |
| 10-19 | 0.3 | 5.0 | 0.006 |
| 20-29 | 1.2 | 5.0 | 0.03 |
| 30-39 | 3.2 | 5.0 | 0.08 |
| 40-49 | 4.9 | 6.3 | 0.15 |
| 50-59 | 10.2 | 12.2 | 0.60 |
| 60-69 | 16.6 | 27.4 | 2.2 |
| 70-79 | 24.3 | 43.2 | 5.1 |
| 80-100 | 27.3 | 70.9 | 9.3 |
