# Supplementary material for: Overview of current state of research on the application of artificial intelligence techniques for COVID-19
Source: PeerJ Comput Sci. 2021 May 26;7:e564. doi: 10.7717/peerj-cs.564 (PMC8176528; doi:10.7717/peerj-cs.564)
Supplement: Supplemental Information 3 [file peerj-cs-07-564-s003.docx]

**Table 3**. Summary of related survey papers

| **Reference** | **Focus of the review** | **Paper source** | **Methodology** | **Number of papers evaluated** |
| --- | --- | --- | --- | --- |
| Albahri et al. [21] | AI and ML applications, systems, algorithms, methods and techniques | IEEE Xplore, Web of Science, PubMed, ScienceDirect, Scopus | PRISMA | 8 |
| Lalmuanawma et al. [22] | Role of AI and ML for screening, predicting, forecasting, contact tracing, and drug development for SARS-CoV-2 | NS* | Convenience sampling | 46 |
| Ozsahin et al. [23] | AI based diagnosis of COVID-19 from chest CT Images | ArXiv, MedRxiv, and Google Scholar | Systematic review | 30 |
| Pham et al. [24] | AI and big data for fighting against COVID-19, | IEEE Xplore, Nature, ScienceDirect, Wiley, arXiv, medRxiv,bioRxiv | Convenience sampling | 144 |
| Rasheed et al. [25] | AI approaches for the diagnosis, anticipate infection and mortality rate by tracing contacts and targeted drug design | Google Scholar | Systematic review | 168 |
| Tseng et al. [26] | Current progress of computational intelligence for fighting COVID-19 | NS* | Convenience sampling | 64 |
| Tayarani [27] | Machine learning techniques for prediction and treatment of COVID-19 infected persons | NS* | Convenience sampling | 654 |

*NS – not specified.
