# Supplementary material for: Overview of current state of research on the application of artificial intelligence techniques for COVID-19
Source: PeerJ Comput Sci. 2021 May 26;7:e564. doi: 10.7717/peerj-cs.564 (PMC8176528; doi:10.7717/peerj-cs.564)
Supplement: Supplemental Information 4 [file peerj-cs-07-564-s004.docx]

**Table 4**. Selection and elimination criterion for shortlisted research articles.

| **Parameter** | **Selection Criteria** | **Elimination Criteria** |
| --- | --- | --- |
| Time Duration | Paper published from 2020-2021 | Paper published before 2020 |
| Investigation | Studies comprising the artificial techniques to predict and diagnosis of COVID-19 | Studies including the genomic and drug concepts related to coronavirus |
| Comparison | Studies concentrate on the surveillance systems and radiological imaging techniques | Studies comprising the existing and repurposing drug design techniques for COVID-19 |
| Imaging Techniques | Studies including the radiological techniques such as CT and X-ray | Studies containing the other than CT and X-ray |
| Design | Studies including experimental results and facts | Studies including Patent, language other than English, case study |
