# Supplementary material for: Overview of current state of research on the application of artificial intelligence techniques for COVID-19
Source: PeerJ Comput Sci. 2021 May 26;7:e564. doi: 10.7717/peerj-cs.564 (PMC8176528; doi:10.7717/peerj-cs.564)
Supplement: Supplemental Information 5 [file peerj-cs-07-564-s005.docx]

**Table 5.** Vigilant systems for COVID-19

| **Name** | **Country** | **Mobile Apps** | **Standalone** | **Technology Used** | **Functionality** |
| --- | --- | --- | --- | --- | --- |
| BlueDot | Hubei | √ | √ | Infectious Disease Vulnerability Index | Health facilities, preventive measures |
| HealthMap | USA | √ | √ | Location Tracking, Bluetooth | Contact tracing |
| RT.live | US | √ | √ | Statistical Models | Health facilities, preventive measures |
| HealthyTogether | Utah | √ | 🗶 | Bluetooth | Symptom tracker, health facilities |
| TraceTogether | Singapore | √ | 🗶 | Bluetooth, Encryption, GPS | Preventive measures, risk assessment, contact tracing |
| Aarogya Setu | India | √ | 🗶 | Bluetooth, GPS | Contact tracing, E-pass, preventive measures |
| COVID Symptom Tracker | USA | √ | 🗶 | Bluetooth, GPS | Contact tracing, preventive measures, risk assessment |
| COVID-19 Quarantine Monitor | India | √ | 🗶 | Bluetooth, Location recording | Symptom tracker, quarantine monitoring |
| COVA Punjab | India | √ | 🗶 | Bluetooth, GPS | Preventive measures, risk assessment, health facilities |
| MP COVID RESPONSE APP | India | √ | 🗶 | Bluetooth | Hotspot identification, peventive measures |
| Quarantine Watch | India | √ | 🗶 | Bluetooth | Quarantine monitoring |
| GoK-Direct Kerala | India | √ | 🗶 | Bluetooth | Preventive measures |
| Test Yourself Goa | India | √ | 🗶 | Bluetooth, chatbot | Self-risk assessment |
| Mahakavach | India | √ | 🗶 | Bluetooth. GPS | Contact tracing, hotspot identification, |
| Haryana Sahayak | India | √ | 🗶 | Map location, Bluetooth | Health facilities, preventive measures, self-risk assessment |
| RajCovidInfo | India | √ | 🗶 | Bluetooth | Preventive measures, Notification of confirmed and death cases |
| Corona Mukt Himachal | India | √ | 🗶 | Bluetooth, GPS | Quarantine monitoring |
| UP Self-Quarantine App | India | √ | 🗶 | Bluetooth, GPS | Quarantine monitoring |
| COVID-19 Odisha | India | √ | 🗶 | Bluetooth, GPS | Hotspot identification, self-risk assessment |
| T COVID 19 | India | √ | 🗶 | GPS, chatbot | Hotspot identification, self-risk assessment, preventive measures |
| Corona-Care | India | √ | 🗶 | Bluetooth, GPS | Symptom tracker, self-risk assessment, quarantine monitoring |
