# Supplementary material for: Overview of current state of research on the application of artificial intelligence techniques for COVID-19
Source: PeerJ Comput Sci. 2021 May 26;7:e564. doi: 10.7717/peerj-cs.564 (PMC8176528; doi:10.7717/peerj-cs.564)
Supplement: Supplemental Information 6 [file peerj-cs-07-564-s006.docx]

**Table 6.** Qualitative analysis of different surveillance mobile apps for COVID-19

| **Name** | **Space requirement (<50MB)** | **Subscription required** | **Advisory** | **Internet required** | **Automatic data entry** |
| --- | --- | --- | --- | --- | --- |
| COVID Symptom Tracker | √ | No | √ | N/A | √ |
| Corona-Care | √ | No | √ | N/A | √ |
| COVID-19 Quarantine Monitor | √ | No | N/A | N/A | √ |
| HealthyTogether | √ | No | √ | N/A | √ |
| TraceTogether | √ | No | √ | N/A | √ |
| Aarogya Setu | √ | No | √ | N/A | √ |
| MP COVID RESPONSE APP | √ | No | 🗶 | N/A | 🗶 |
| Quarantine Watch | √ | N/A | 🗶 | N/A | 🗶 |
| COVA Punjab | √ | No | √ | N/A | √ |
| Mahakavach | √ | No | √ | N/A | √ |

N/A-Not Applicable
