# Supplementary material for: Overview of current state of research on the application of artificial intelligence techniques for COVID-19
Source: PeerJ Comput Sci. 2021 May 26;7:e564. doi: 10.7717/peerj-cs.564 (PMC8176528; doi:10.7717/peerj-cs.564)
Supplement: Supplemental Information 7 [file peerj-cs-07-564-s007.docx]

**Table 7.** Comparison of Data Visualization Systems

| **Dashboard Name** | **Country** | **Infected cases** | **Recovered Cases** | **Death cases** | **Test Conducted** | **Discharge** | **Daily Change** |
| --- | --- | --- | --- | --- | --- | --- | --- |
| JHU-CSSE | Global | √ | √ | √ | √ | 🗶 | 🗶 |
| WHO COVID-19 | Global | √ | √ | √ | √ | 🗶 | √ |
| SharedGeo | Global | √ | √ | √ | 🗶 | 🗶 | √ |
| Thebaselab | Global | √ | √ | √ | 🗶 | 🗶 | 🗶 |
| NextStrain | Global | √ | √ | √ | 🗶 | 🗶 | 🗶 |
| BBC | Global | √ | √ | √ | 🗶 | 🗶 | 🗶 |
| New York Times | Global | √ | √ | √ | 🗶 | 🗶 | 🗶 |
| HealthMap | Global | √ | √ | √ | 🗶 | 🗶 | 🗶 |
| Bing’s AI Tracker | Global | √ | √ | √ | 🗶 | 🗶 | 🗶 |
| COVID-19 Data Hub | Global | √ | √ | √ | 🗶 | √ | √ |
| COVID-19 ZA South Africa | South Africa | √ | √ | √ | √ | √ | √ |
| COVID19 India | India | √ | √ | √ | √ | 🗶 | 🗶 |
| Worldometer | Global | √ | √ | √ | √ | √ | √ |
| South China Morning Post | Global | √ | √ | √ | 🗶 | 🗶 | 🗶 |
| CDC | USA | √ | √ | √ | 🗶 | 🗶 | 🗶 |
| COVID Tracking | USA | √ | √ | √ | √ | 🗶 | 🗶 |
| COVID Act Now | USA | √ | √ | √ | √ | √ | √ |
| University of Virginia COVID-19 | Global | √ | √ | √ | √ | √ | √ |
