# Supplementary material for: Overview of current state of research on the application of artificial intelligence techniques for COVID-19
Source: PeerJ Comput Sci. 2021 May 26;7:e564. doi: 10.7717/peerj-cs.564 (PMC8176528; doi:10.7717/peerj-cs.564)
Supplement: Supplemental Information 8 [file peerj-cs-07-564-s008.docx]

**Table 8.** Description of COVID-19 datasets and resources

| **Type** | **Dataset Name** | **Downloadable Link** | **Description** |
| --- | --- | --- | --- |
| Text | WHO Global Research Database | https://www.who.int/gho/database/en/ | Repository of COVID-19 articles, reports |
|  | COVID-19 Open Search Dataset | https://www.semanticscholar.org/cord19 | 2,80,000 articles |
|  | LitCOVID | go.nature.com/3almd5p | 1,19,472 articles |
|  | AI COVID-19 | https://covid-19.dimensions.ai | Repository of 13,959 datasets |
| Social Media | COVID-19 Twitter | https://github.com/thepanacealab/covid19_twitter | 1 billion Tweets |
|  | COVID-19 TweetIDs | https://github.com/echen102/COVID-19-TweetIDs | 1,359,591,254 Tweets |
|  | COVID-19 Tweets | https://www.kaggle.com/smid80/coronavirus-covid19-tweets | 500,000 Tweets |
|  | COVID-19 Real World Worry | https://github.com/ben-aaron188/covid19worry | 5,000 texts, emotions measuring |
|  | COVID-19 Coronavirus News Article | https://www.covid19-archive.com/ | - |
|  | COVID-19 Television Coverage | https://blog.gdeltproject.org/a-new-dataset-for-exploring-the-coronavirus-narrative-on-television-news/ | 119,083 mentions, each mention has 15 second clip |
| Biomedical | Chest X-Ray Dataset | https://www.kaggle.com/bachrr/covid-chest-xray | 127 COVID-19, 127 pneumonia, and 127 healthy images |
|  | COVID-19 Survival Calculator | https://www.covid19survivalcalculator.com/calculator | 17 million adult patients |
|  | United Against Covid | http://united-against-covid.org/ | - |
|  | COVID-19 Data Clearinghouse | https://asone.ai/polymath/index.php?title=COVID-19_dataset_clearinghouse | Repository of public COVID-19 datasets |
|  | Global Health Drug Discovery Institute | https://ghddi-ailab.github.io/Targeting2019-nCoV/ | 12,000 drugs, 3D structure data |
|  | COVID-19 antiviral Compounds | https://www.cas.org/covid-19-antiviral-compounds-dataset | 50,000 chemical substances, SD file format |
|  | Italian Society of Medical and Interventional Radiology data set | https://www.kaggle.com/paultimothymooney/chest-xray-pneumonia | 190 COVID-19, 1345 viral pneumonia, 1341 normal images |
|  | Point-of-care Ultrasound dataset | https://github.com/jannisborn/covid19_ultrasound | 654 COVID-19, 277 bacterial pneumonia, and 172 healthy images |
| Speech | COVID-19 sound | https://www.covid-19-sounds.org/en/ | 7000 sample size |
|  | Coswara-Dat | https://github.com/iiscleap/Coswara-Dat | 1000 sample size |
|  | Virufy-covid | https://github.com/virufy/virufy-covid | 16 sample size |
| Case Study | CHIME | https://chimechallenge.github.io/chime6/ | - |
|  | Github | https://datahub.io/core/covid-19 | 118,000 samples, time series data |
|  | Kaggle | https://www.kaggle.com/datasets | - |
|  | Johns Hopkins CSSE | https://github.com/CSSEGISandData/COVID-19 | - |
|  | Humanitarian Data Exchange | https://data.humdata.org/event/covid-19 | - |
|  | H_2_O.ai | https://www.h2o.ai/covid-19/ | - |
|  | Online Server | http://biomed.nscc-gz.cn/server/Ncov2019 | - |
|  | COVID Scholar | https://covidscholar.org | - |
