# Supplementary material for: Overview of current state of research on the application of artificial intelligence techniques for COVID-19
Source: PeerJ Comput Sci. 2021 May 26;7:e564. doi: 10.7717/peerj-cs.564 (PMC8176528; doi:10.7717/peerj-cs.564)
Supplement: Supplemental Information 9 [file peerj-cs-07-564-s009.docx]

**Table 9.** Comparative analysis of prediction models

| **Ref.** | **Model used** | **Assumptions** | **Dataset/source** | **Software used** | **Performance** |
| --- | --- | --- | --- | --- | --- |
| [49] | XGBoost classifier | No assumption | 2,779 suspected patients | Python | 90% prediction accuracy |
| [50] | SVM | No assumption | 53 patients | Python | 80% prediction accuracy |
| [51] | SVM, ANN, Random forest regression | No assumption | 80 patients | MATLAB R2017a | 90% prediction accuracy |
| [52] | SEIR | Incubation period=5.1, Infectious period=7, Growth rate of epidemic=1.15 | 1375.98 million patients | MATLAB 2018b, SimVoi | Death rate and hospitalization are reduced by 90% |
| [53] | Multiple and linear regression | No assumption | covid19india.org. | Python, excel with XL-STAT | Predicted death rate is 211 at the end of 5^th^ week |
| [54] | Deep transfer learning | No assumption | 3-second cough samples | Python | 92.85% prediction accuracy |
| [55] | LASSO regression | Median age of patients>13 years | 164 patients | - | 84.1% prediction accuracy |
| [56] | Machine learning algorithm | Travel history within 14 days | - | - | Spread of virus can be reduced by 10% |
| [57] | Random forest | Median age of patients=46.5±16.5 years | 118 patients | Python | 89% prediction accuracy |
| [58] | Logistic regression, Random forest | Hospital stay=10 days | 58 patients, 135 lesions | Python, 3Dslicer | 90% prediction accuracy |
| [59] | SEIR | Incubation period=5, Infectious period=7 | World meters | Python | Reduction in infection due to social distancing is 78% |
