# Supplementary material for: Overview of current state of research on the application of artificial intelligence techniques for COVID-19
Source: PeerJ Comput Sci. 2021 May 26;7:e564. doi: 10.7717/peerj-cs.564 (PMC8176528; doi:10.7717/peerj-cs.564)
Supplement: Supplemental Information 10 [file peerj-cs-07-564-s010.docx]

**Table 10.** Artificial Intelligence Tools/Techniques for screening of COVID-19 symptoms

|  | **Tools used** | **Company/Organization** | **Mechanism** |
| --- | --- | --- | --- |
| Face Recognition | Infrared Camera | Chinese firm Baidu | Body Temperature |
| Wearable Device | OURA Ring | OURA | Body Temperature, Heart Rate |
|  | Apple Watch | Apple | Body Temperature, Heart Rate |
|  | Halo Wrist Watch | Proxxi technologies | Bluetooth for Physical distancing (6 feet) |
|  | Whoop Wearable Band | WHOOP | Respiratory Rate |
| Virtual Assistant | WhatsApp Bot | Facebook | Question-Answering |
|  | Stallion Bilingual Chatbot | Stallion.AI | Image Analysis and Interpretation System |
|  | Microsoft’s Healthcare Bot | Microsoft | Interactive voice and text |
|  | Zoe Chatbot | South Australian | Question-Answering |
|  | Google Cloud | Google | Google Chat’s Widget |
|  | AskDoc | BITS | Question-Answering |
