# Supplementary material for: Overview of current state of research on the application of artificial intelligence techniques for COVID-19
Source: PeerJ Comput Sci. 2021 May 26;7:e564. doi: 10.7717/peerj-cs.564 (PMC8176528; doi:10.7717/peerj-cs.564)
Supplement: Supplemental Information 11 [file peerj-cs-07-564-s011.docx]

**Table 11.** AI tools for diagnosis of COVID-19

| **Developer** | **Tool/Software** | **Software** | **Hardware** | **Mechanism** |
| --- | --- | --- | --- | --- |
| Baidu’s team | LinerFold | √ |  | Prediction time reduced to 27 seconds |
| China | InferVISION | √ |  | AI-powered Medical Imaging |
| Shenzhen | MicroMultiCopter |  | √ | Carry medical samples from congested areas |
| DarwinAI | COVID-Net | √ |  | Predict COVID infection in Chest X-ray |
| Mumbai-based Qure.ai | qXR | √ |  | AI-based Medical Imaging |
| Lunit | INSIGHT CXR | √ |  | AI-power chest X-rays Analysis |
| Thirona and Delft Imaging | CAD4COVID | √ |  | Detection of abnormalities in Lung |
| Danish | UVD Robots |  | √ | Disinfect the infected rooms and communication |
| Stanford University | Epic Model | √ |  | Predict whether the patient has to shift in ICU or not |
| Bayesian Health Startup in JHU | Respiratory Model | √ |  | Predict whether the patient is deteriorate in coming hours or not |
| Rutgers University and Robert Wood Johnson University Hospital | Automated Venipuncture Device |  | √ | Take blood samples from patients. Success rate is 87% |
| RoboEarth | Humanoid serving robot (Amigo) |  | √ | Housekeeping operations for patients |
| Heriot-Watt University | Socially Assistive Robots |  | √ | Conversation with elders to relieve the stress |
| Chinese Government | Lockdown patrol UAVs |  | √ | Assist law enforcement and warning people during pandemic lockdown |
