# Supplementary material for: Overview of current state of research on the application of artificial intelligence techniques for COVID-19
Source: PeerJ Comput Sci. 2021 May 26;7:e564. doi: 10.7717/peerj-cs.564 (PMC8176528; doi:10.7717/peerj-cs.564)
Supplement: Supplemental Information 12 [file peerj-cs-07-564-s012.docx]

**Table 12.** Deep learning/Machine learning techniques used for Chest X-ray images

| **Ref.** | **Models used** | **No. of cases** | **Performance Measures** | | | | | |
| --- | --- | --- | --- | --- | --- | --- | --- | --- |
|  |  |  | **Accuracy** | **Precision** | **Sensitivity** | **Specificity** | **AUC** | **F-score** |
| [90] | VGG19, DenseNet121, ResNetV2, InceptionV3, InceptionResNetV2, Xception, MobileNet | 25 positive, 25 Normal | 90% | 0.83 | - | - | 0.90 | 0.91 |
| [91] | COVID-Net | 53 positive, 5526 negative, 8066 Healthy | 93.3% | 0.91 | - | - | - | - |
| [92] | VGG19 | 224 positive, 700 Pneumonia, 504 Healthy | 96.78% | - | 98.66% | 96.46% | - | - |
| [93] | CoroNet | 157 positive, 500 Pneumonia, 500 Healthy | 89.6% | 0.93 | 95.3% | 96.4% | - | 0.95 |
| [94] | DarkCovidNet | 125 positive, 500 Pneumonia, 500 Healthy | 87.02% | 0.89 | 85.35% | 92.18% | - | 0.87 |
| [95] | ResNet50, SVM | 25 positive, 25 negative | 95.38% | - | 95.33% | - | - | 0.95 |
| [96] | AlexNet, SPEA-II | 100 positive, 134 Pneumonia, 155 Healthy. 188 tuberculosis | 99.13% | - | 99.47% | 99.15% | 0.99 | - |
| [97] | Inception V3, Xception Net, ResNeXt | 576 positive, 4273 Pneumonia, 1583 Healthy | 97.97% | 0.99 | - | - | - | 0.95 |
| [98] | VGG19, InceptionV3, CheXNet, ResNet, MobileNet, SqueezeNet | 424 positive, 1485 Pneumonia, 1579 Healthy | 99.7% | 0.99 | 99.7% | 99.55% | - | - |
| [99] | LSTM | 613 positive, 1525 Pneumonia, 1525 Healthy | 99.4% | - | 99.3% | 99.2% | 0.99 | 0.98 |
| [100] | CNN, SVM | 219 positive, 1345 Pneumonia, 1341 Healthy | 98.97% | - | 89.39% | 99.75% | - | 0.96 |
| [101] | Ensemble Resnet18 | 180 positive, 74 Pneumonia, 57 tuberculosis, 191 Healthy | 88.9% | 0.83 | - | 96.4% | - | 0.84 |
| [102] | InceptionV3 | 180 positive, 22 MERS, 22 SARS, 22 pneumonia, 20 varicella, 2000 Healthy | - | - | - | - | - | 0.89 |
| [103] | Manta-ray foraging, KNN | 216 positive, 1675 healthy | 96.09% | 0.98 | - | - | - | - |
| [104] | ResNet50, ResNet101 | 440 positive, 937 Pneumonia, 455 Healthy | 98.93% | 0.98 | 98.93% | 98.66% | - | 0.98 |
| [105] | nCOVNet | 192 positive, 5863 Healthy | 88.09% | - | 97.62% | 78.57% | - | - |
| [106] | InceptionV3 | 122 positive, 300 pneumonia, 150 Healthy | 85% | - | 94% | 92.7% | 0.96 | - |
| [107] | VGG16, DenseNet | 401 positive, 401 pneumonia, 401 Healthy | 99.21% | 0.99 | - | - | - | 0.99 |
| [108] | CNN, MADE | 127 positive, 500 Pneumonia, 500 Healthy | 94.48% | - | 93.83% | 93.83% | - | - |
| [109] | Xception | 127 positive, 500 Pneumonia, 500 Healthy | 97.40% | - | 97.09% | 97.29% | - | - |
| [110] | 2D curvelet transform, chaotic salp swarm, deep learning | 2660 positive, 2660 pneumonia, 2660 Healthy | 99.69% | 0.99 | - | 99.44% | - | - |
