# Supplementary material for: Overview of current state of research on the application of artificial intelligence techniques for COVID-19
Source: PeerJ Comput Sci. 2021 May 26;7:e564. doi: 10.7717/peerj-cs.564 (PMC8176528; doi:10.7717/peerj-cs.564)
Supplement: Supplemental Information 13 [file peerj-cs-07-564-s013.docx]

**Table 13.** Deep learning/Machine learning techniques used for Chest CT-scan images

| **Ref.** | **Models used** | **No. of cases** | **Performance Measures** | | | | | |
| --- | --- | --- | --- | --- | --- | --- | --- | --- |
|  |  |  | **Accuracy** | **Precision** | **Sensitivity** | **Specificity** | **AUC** | **F-score** |
| [113] | InceptionV3 | 1065 CT images | 89.5% | - | 87% | 88% | - | 0.77 |
| [114] | SRGAN, VGG16 | 275 positive, 195 healthy | 98.0% | - | 99% | 94.9% | - | - |
| [115] | ResNet18 | 90 CT images | 86.7% | 0.86 | - | - | - | 0.84 |
| [116] | CNN, MODE | 126 CT images | 93.5% | - | 90.5% | 90.8% | - | - |
| [117] | COVNet | 4356 CT images | - | - | 90% | 96% | 0.96 | - |
| [118] | CoroNet | 905 CT images | - | - | 84.3% | 82.8% | 0.92 | - |
| [119] | LSTM | 321 CT images | 99.68% | - | - | - | - | - |
| [120] | ResNet50 | 495 CT images | 81.9% | - | 76% | 81.1% | - | - |
| [121] | ResNet50 | 3993 CT images | 99.87% | - | 99.58% | 100% | - | - |
| [122] | DenseNet201 | 2492 CT images | 96.25% | 0.96 | - | 96.21% | 0.97 | - |
| [123] | DCCN, ResNet152, VGG16 | 2373 positive, 2890 pneumonia, 3193 tuberculosis, 3038 healthy | 98.83% | - | 98.83% | 98.82% | 0.98 | - |
