# Supplementary material for: Overview of current state of research on the application of artificial intelligence techniques for COVID-19
Source: PeerJ Comput Sci. 2021 May 26;7:e564. doi: 10.7717/peerj-cs.564 (PMC8176528; doi:10.7717/peerj-cs.564)
Supplement: Supplemental Information 14 [file peerj-cs-07-564-s014.docx]

**Table 14.** Impact of rumors and loathe speech on Social Life

|  | **Technique** | **Methods** | **Mechanism** |
| --- | --- | --- | --- |
| Social Media | Galotti et al. [147] | Inodemic Risk Index | Impact of infodemics |
|  | Cinelli et al. [148] | ML Models | Analysis of comments, likes, and reactions |
|  | Mejova et al. [149] | Facebook Ad Library | Analysis of advertisements |
|  | Zarocosta [150] | AI tool | Stop the COVID-19 rumors |
|  | Pandey et al. [151] | AI and NLP | Verification of sources |
|  | WHO multilingual Chat  [152] | Virtual Assistant | Analysis of spreading sources |
| Loathe Speech | Velasquez et al. [153] | ML Models | Non-Verbal Behaviors |
|  | Schild et al. [154] | ML Models | Sinophopic Behaviors of tweets |
